# A case of Extrapulmonary intrathoracic hydatidosis with pseudochylothorax

**DOI:** 10.1002/ccr3.1628

**Published:** 2018-06-19

**Authors:** Shahideh Amini, Zohreh Kahramfar, Besharat Rahimi

**Affiliations:** ^1^ Clinical Pharmacy Department Faculty of Pharmacy Tehran University of Medical Sciences Tehran Iran; ^2^ Department of Pulmonary Medicine Faculty of Medicine Tehran University of Medical Sciences Tehran Iran

**Keywords:** infectious diseases, pharmacology, respiratory medicine

## Abstract

*Echinococcus* is a great re‐emerging public health issue. Intrathoracic and extra pulmonary hydatid cysts with pseudochylothorax are rare. There is no standard treatment in case of hydatidosis with pseudochylothorax. Pharmacotherapy approaches may be an option in case of long duration of disease and high risk for surgery.

## 1 INTRODUCTION

Hydatid disease in men is caused by infection with the larval stage of the dog tapeworm *Echinococcus granulosus*. It is followed by an accidental ingestion of tapeworm eggs extracted in the feces of infected dogs. The overall prevalence of echinococcal infection is increasing. The prevalence of liver and pulmonary hydatid cysts increases with age.^1^ Hydatid cysts may be found in almost any site of the body, the liver and lung are affected more than other organs.^1^ *Echinococcus* produces a wide spectrum of diseases with different clinical presentations. The parasite load, the site and the size of cysts determine the degree of symptoms.^1^, ^2^

The most common clinical features of lung hydatid cyst are cough, chest pain, dyspnea, and hemoptysis. The cyst may be complicated by infection or rupture to bronchial tree or the pleural cavity.^3^, ^4^ Pseudochylothorax was rarely seen.^5^

There is no “best” treatment option for echinococcal infection; percutaneous treatments, surgery, antiparasitic treatment and watch and wait are available different treatment modalities.^6^, ^7^, ^8^

## 2 CASE PRESENTATION

A 79‐year‐old Iranian man was admitted to Imam‐Khomeini hospital with 6‐month history of dyspnea, nonproductive cough, fatigue and weight loss. Previous clinical history and family history was unremarkable. He had no history of smoking or being passive smoker. He did not use alcohol or opium in the past, and his job is animal husbandry. Six month before recent admission, he had been admitted into another hospital, due to dyspnea. His spiral thoracic computed tomography scan revealed left massive pleural effusion with multiple cysts inside pleural effusion. The patient refused more investigation and discharged with his satisfaction. From first to second hospitalization, a little relief of his symptoms was seen. On February 2017, the patient was admitted again and his complaint was dyspnea and dry cough.

General physical examination revealed an oral temperature of 37°C, a heart rate of 90 beats per minute, a respiratory rate of 14 breaths per minute, and a blood pressure of 110/70 mm Hg and the oxygen saturation of 92% with room air. Aside from this, the clinical examination was unremarkable. Blood samples revealed an elevated CRP of 97 mg/L, leukocytosis (15400/microL) with a strong component of granulocytosis, but no eosinophilia. Physical chest examination showed decreased breath sound in left hemithorax. The remainder of the examination was normal.

**Figure 1:**
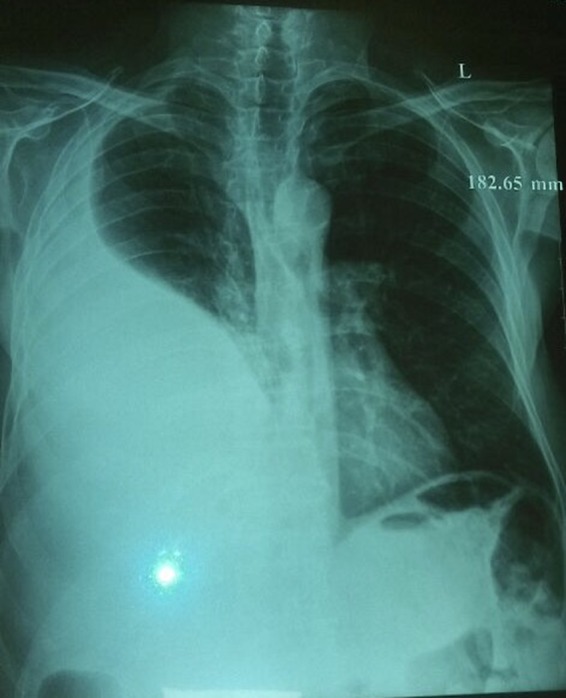
AP view of CXR showed pleural effusion

**Figure 2:**
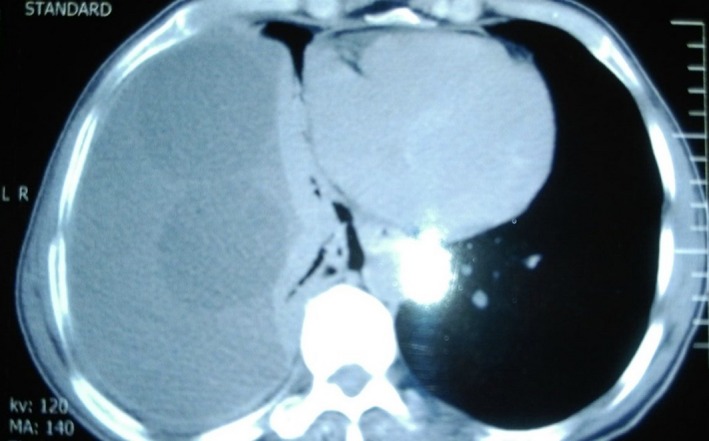
Mediastinal view of lung CT scan shows massive pleural effusion and multiple intra pleural hydatid cysts

**Figure 3:**
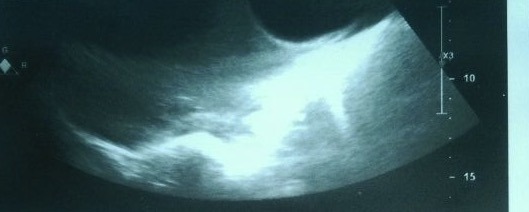
Right pleural sonography shows ruptured hydatid cysts

**Figure 4:**
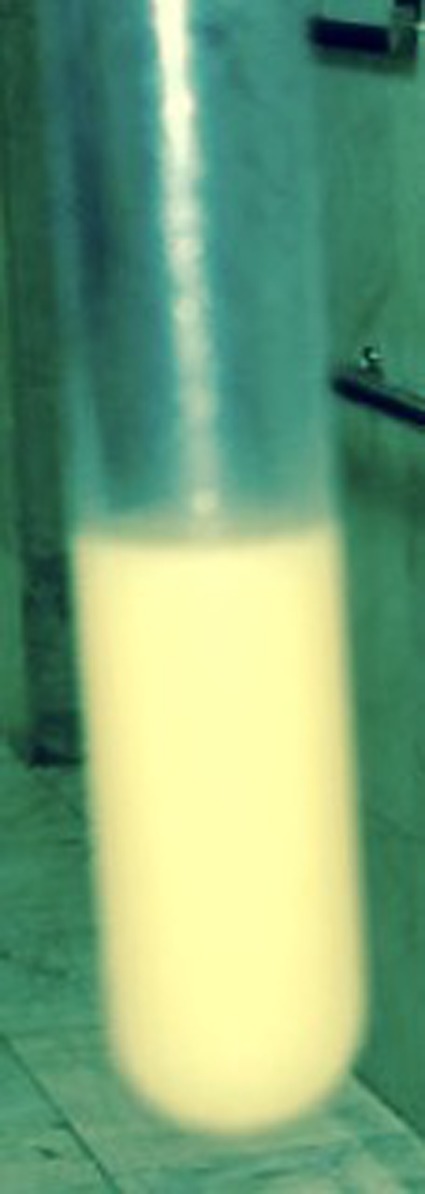
Thoracentesis of pleural effusion showed a milky fluid

The chest radiography showed left pleural effusion. Spiral thoracic computed tomography scan revealed left loculated pleural effusion (Figures 1 and 2). Echocardiography was normal and abdominal sonography revealed one cyst with 59 mm diameter and many wrinkle membranes in pleural space (Figure 3). Thoracentesis obtained a milky fluid (Figure 4) with the following biochemical values: 4800 leukocytes/mm^3^ with polymorphonuclear predominancy leukocytes/mm^3^; total protein 10.8 g/dL; lactate dehydrogenase (LDH) 5744 U/L; glucose 81 mg/dL; pH 7.34; Cholesterol 189 mg/dL triglycerides 68 mg/dL. A great number of cholesterol crystals were observed on microscopic examination. H & E stain of pleural fluid showed many scolices of hydatid cyst. Immunoglobin‐G enzyme‐linked immunosorbent assay for *Echinococcus* was positive, and then pseudochylothorax pleural effusion and spontaneously ruptured hydatid cyst was diagnosed.

The patient declined any surgical treatment; therefore he was prescribed antihelminthic treatment with albendazole 400 mg, two times daily. He was discharged with satisfaction. After 9 months follow up dyspnea was improved and the pleural effusion resolved gradually. After this time albendazole was discounted.

## 3 DISCUSSION

In this case, we described a 79‐year‐old man with complaint of dyspnea caused by pseudochylothorax pleural effusion and spontaneously ruptured hydatid cyst. Rupture of a hydatid cyst into the pleural cavity usually causes pneumothorax, pleural effusion, empyema and allergic or anaphylactic reaction.^9^, ^10^ Unlike the literature, the only symptom, in this case, was dyspnea and pleural effusion. Our explanation for no symptom of cyst rupture was weaker immune response and elderly age in this case.

On the other hand, pseudochylothoraces has been seen in this case. Pseudochylothoraces or cholesterol effusions typically develop in the setting of chronic pleural inflammation and characteristically persists over time.^5^, ^11^ *Echinococcosis* is rare causes of cholesterol effusions that occurred in this case.

According to our review, intrathoracic and extra pulmonary hydatid cysts are rare and can be located in the fissures, pleural cavity, chest wall, mediastinum, myocardium, and diaphragm. Primary pleural hydatid cysts are extremely rare.^12^

CT scanning is the main diagnostic tool. On CT scan simple hydatid cysts have water density with thin border that may be able to calcify. In our case, two cysts were found in pleural space without calcified border.

In our case at his first hospitalization investigation and more diagnostic evaluation were not done but at his second hospitalization we faced interapleural, ruptured membranes of cysts on ulterasonography and cholesterol effusions according to pleural fluid analysis was diagnosed.

The effective treatment of hydatid cysts in organs especially in lungs is complete excision of cysts. Additional medical treatment with Albendazole should be carried for high‐risk group patients.^13^ There is no standard treatment in case of hydatidosis with pseudochylothorax. In case of cholesterol effusions due to paragonimiasis, successful treatment of it has been reported with praziquantel.^13^ In this case, the patient refused any surgical treatment, so albendazole 400 mg, two times a day, was administrated for 9 months. Regarding the occurrence of cholesterol effusions that indicated to chronicity of disease and overall symptom that has not changed over time, the decision was made to prescribed oral treatment.

To the best our knowledge, this the first report of primary and complicated hydatid cysts with so high pleural protein and LDH with pseudochylothoraces and without any symptom of systemic infection that respond to albendazole. In the future, other studies can explain more about the best treatment for patient with long duration of disease or hydatidosis with pseudochylothorax.

## CONFLICT OF INTEREST

None declared.

## AUTHORSHIP

SA: is the guarantor of the manuscript, taking responsibility for the integrity of the work as a whole, from inception to published article. ZK and BR: aggregated the data, created the figures, and helped draft the discussion of the manuscript.

## References

[1] Jenkins DJ , Romig T , Thompson RC . Emergence/re‐emergence of *Echinococcus* spp. a global update. Int J Parasitol. 2005;35:1205‐1219. 16157340 10.1016/j.ijpara.2005.07.014

[2] Dziri C , Haouet K , Fingerhut A , Zaouche A . Management of cystic echinococcosis complications and dissemination: where is the evidence? World J Surg. 2009;33:1266. 19350321 10.1007/s00268-009-9982-9

[3] Santivanez S , Garcia HH . Pulmonary cystic echinococcosis. Curr Opin Pulm Med. 2010;16:257‐261. 20216420 10.1097/MCP.0b013e3283386282 PMC3362862

[4] Arinc S , Kosif A , Ertugrul M , et al. Evaluation of pulmonary hydatid cyst cases. Int J Surg. 2009;7:192‐195. 19369124 10.1016/j.ijsu.2008.11.003

[5] Lama A , Ferreiro L , Toubes ME , et al. Characteristics of patients with pseudochylothorax—a systematic review. J Thorac Dis. 2016;8:2093‐2101. 27621864 10.21037/jtd.2016.07.84 PMC4999702

[6] Brunetti E , Kern P , Vuitton DA ; Writing Panel for the WHO‐IWGE . Expert consensus for the diagnosis and treatment of cystic and alveolar echinococcosis in humans. Acta Trop. 2010;114:1‐16. 19931502 10.1016/j.actatropica.2009.11.001

[7] Ulger S , Barut H , Tunc M , et al. Radiation therapy for resistant sternal hydatid disease. Strahlenther Onkol. 2013;189:508‐509. 23604185 10.1007/s00066-013-0322-5

[8] Neumayr A , Troia G , Bernardis C , et al. Justified concern or exaggerated fear: the risk of cystic echinococcosis a systemic literature review. PLOS Negl Trop Dis. 2011;5:e1154. 21695106 10.1371/journal.pntd.0001154 PMC3114754

[9] Shameem M , Akhtar J , Bhargava R , et al. Ruptured pulmonary hydatid cyst with anaphylactic shock and pneumothorax. Respir Care. 2011;56:863‐865. 21333077 10.4187/respcare.00821

[10] Turgut AT , Altinok T , Topçu S , Koşar U . Local complications of hydatid disease involving thoracic cavity: imaging findings. Eur J Radiol. 2009;70:49‐56. 18291609 10.1016/j.ejrad.2008.01.002

[11] Ozvaran MK , Ersoy Y , Uskul B , et al. Pleural complications of pulmonary hydatid disease. Respirology. 2004;9:115‐119. 14982612 10.1111/j.1440-1843.2003.00518.x

[12] Gursoy S , Ucvet A , Tozum H , Erbaycu AE , Kul C , Basok O . Primary intrathoracic extrapulmonary hydatid cysts: analysis of 14 patients with a rare clinical entity. Tex Heart Inst J. 2009;36:230‐233. 19568393 PMC2696497

[13] Srinivasan B , Mohite PN , Thingnam SK . Extrapulmonary intrapleural hydatid cysts‐rare variant of uncommon disease. Indian J Thorac Cardiovasc Surg. 2010;26:247‐250.

